# Chitooligosaccharides accelarate myelin clearance by Wipi1 mediated Schwann cell autophagy promoting peripheral nerve regeneration

**DOI:** 10.1093/rb/rbaf044

**Published:** 2025-05-19

**Authors:** Hongkui Wang, Miao Zhang, Mengke Liu, Jina Liu, Jiahuan Gong, Long Yin, Yumin Yang, Yahong Zhao

**Affiliations:** Key Laboratory of Neuroregeneration of Jiangsu and Ministry of Education, Co-innovation Center of Neuroregeneration, NMPA Key Laboratory for Research and Evaluation of Tissue Engineering Technology Products, Nantong University, Nantong 226001, P. R. China; Key Laboratory of Neuroregeneration of Jiangsu and Ministry of Education, Co-innovation Center of Neuroregeneration, NMPA Key Laboratory for Research and Evaluation of Tissue Engineering Technology Products, Nantong University, Nantong 226001, P. R. China; Key Laboratory of Neuroregeneration of Jiangsu and Ministry of Education, Co-innovation Center of Neuroregeneration, NMPA Key Laboratory for Research and Evaluation of Tissue Engineering Technology Products, Nantong University, Nantong 226001, P. R. China; Key Laboratory of Neuroregeneration of Jiangsu and Ministry of Education, Co-innovation Center of Neuroregeneration, NMPA Key Laboratory for Research and Evaluation of Tissue Engineering Technology Products, Nantong University, Nantong 226001, P. R. China; Key Laboratory of Neuroregeneration of Jiangsu and Ministry of Education, Co-innovation Center of Neuroregeneration, NMPA Key Laboratory for Research and Evaluation of Tissue Engineering Technology Products, Nantong University, Nantong 226001, P. R. China; Key Laboratory of Neuroregeneration of Jiangsu and Ministry of Education, Co-innovation Center of Neuroregeneration, NMPA Key Laboratory for Research and Evaluation of Tissue Engineering Technology Products, Nantong University, Nantong 226001, P. R. China; Key Laboratory of Neuroregeneration of Jiangsu and Ministry of Education, Co-innovation Center of Neuroregeneration, NMPA Key Laboratory for Research and Evaluation of Tissue Engineering Technology Products, Nantong University, Nantong 226001, P. R. China; Key Laboratory of Neuroregeneration of Jiangsu and Ministry of Education, Co-innovation Center of Neuroregeneration, NMPA Key Laboratory for Research and Evaluation of Tissue Engineering Technology Products, Nantong University, Nantong 226001, P. R. China

**Keywords:** chitooligosaccharide, myelin clearance, Schwann cell, autophagy, Wipi1

## Abstract

As the most feasible method to reconstruct long-distance peripheral nerve injuries, tissue-engineered nerves rely on biomaterials as a key driving factor. Chitooligosaccharides, intermediate products of chitosan degradation, have the ability to positively regulate nerve regeneration microenvironments. However, the impact of chitooligosaccharides on clearance of myelin debris during Wallerian degeneration is unrevealed. The focus is on exploring the role of chitooligosaccharides in myelin clearance, which is a crucial preparation stage for nerve regeneration. The effects of chitooligosaccharides on nerve regeneration were demonstrated through the morphological and functional evaluations. Then, the myelin lipids and proteins were analyzed using the morphological staining, and molecular and protein detection. The microstructure and ultrastructure observations of lysosomes and autophagosomes were performed. In addition, the proteomics and bioinformatics analysis of injured nerves treated with chitooligosaccharides. The interacting molecules and the regulatory network of Wipi1 were further predicted. On the basis of positive roles on peripheral nerve regeneration, it was illustrated that chitooligosaccharides accelerated the clearance of myelin. Furthermore chitooligosaccharides could regulate lysosomal and autophagic functions, and its role in promoting myelin clearance was mainly related to the enhanced autophagy of Schwann cells rather than macrophages. The big data analysis revealed that Wipi1 was notably upregulated in Schwann cells, mediating chitooligosaccharides to promote autophagy and myelin clearance. Meanwhile, as a potential therapeutic target, Wipi1 significantly accelerated myelin clearance and lipid metabolism after peripheral nerve injury. Our research deepens the comprehensive understanding of the positive regulatory role of chitosan and chitooligosaccharides; and it expands new content and ideas for designing and constructing better tissue-engineered nerves from the perspective of mutual communication and response between biomaterials and body tissues.

## Graphical abstract

**Figure:**
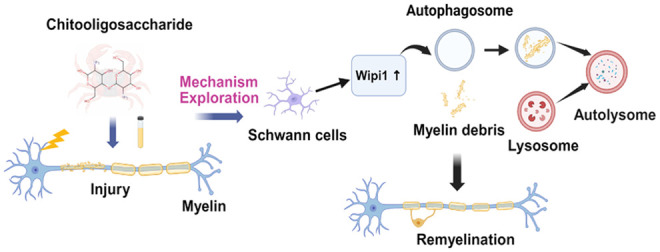

## Introduction

The peripheral nervous system possesses certain self-healing capacity, and its functional recovery is related to the quality of local nerve regeneration microenvironment [1–3]. The peripheral nerve regeneration microenvironment refers to all factors that affect nerve regeneration in terms of time and space in the regeneration chamber involving the communications between biomaterials and tissues [4–6], such as vascularization, inflammation and immunity, chemotaxis and adhesion and guidance to cells. In addition, the sufficient growth space is required first for peripheral nerves regeneration. Wallerian degeneration is quickly initiated in the distal nerve trunk post lesion [7, 8]. The cytoskeletons disintegrate, axons collapse into granular and amorphous fragments, and myelin sheaths disintegrate to a large number of fragments [9, 10]. Meanwhile, accompanied by migration and aggregation of Schwann cells (SCs) and macrophages, damaged axons and myelin debris are degraded and cleared [11–13]. This is precisely to leave sufficient space in advance for nerve regeneration, so that subsequent biological processes such as axonal growth and myelin formation can proceed normally.

Therefore, myelin clearance is crucial for nerve regeneration. The rupture of myelin after nerve injury leads to the production of myelin ovoid fragments, which contain many factors including myelin associated glycoprotein (MAG). These factors restrain axonal regeneration and hinder the process of nerve repair [14, 15]. For the peripheral nervous system, the rapid cellular response enables the rapid myelin clearance, creating a favorable extracellular environment that promotes axonal regeneration. If myelin debris in the early stage of injury cannot be cleared in time, it will hinder axonal regeneration. Studies have shown that the weak regenerative constitution of central nervous system is mainly due to slow cellular responses [16, 17], resulting in delayed clearance of tissue debris [18, 19]. Researches demonstrated that clearance of axon and myelin debris is mainly regulated by the joint participation of SCs and macrophages [14, 20, 21]. After peripheral nerve injury, macrophages polarize and exhibit phagocytic activity. Macrophages are recruited hours to days after injury, and infiltrating macrophages are filled with myelin derived fat, which are the main cells for clearing debris [22]. The important role of macrophages in myelin clearance has been fully demonstrated, but the mechanisms by which SCs participate in myelin breakdown remains unclear. It has been confirmed that autophagy of SCs is involved in regulating the pathological and physiological changes after peripheral nerve injury, and researches on its specific mechanisms is constantly deepening [20, 23]. The autophagy of SCs is not only a precursor to the myelin clearance but also has a regulatory effect on macrophages [24, 25], playing a dominant role in the clearance process of myelin debris.

Autologous nerve transplant remains the gold standard of peripheral nerve repair. However, tissue engineered nerves are currently the best choice for the long defect repair. There is still much exploration space for improving the local nerve regeneration microenvironment [26]. Biomaterials are the core factor driving the continuous research and application of tissue engineering nerves forward. Biomaterials first serve as scaffolds for tissue regeneration, simulating various properties of natural tissue matrices, especially mechanical properties such as strength and elasticity, to provide a better mechanical basis for reconstructing the regeneration microenvironment [27, 28]. The mechanical properties of biomaterials can directly trigger biological responses in tissues and cells, thereby converting mechanical signals into chemical and biological signals [29, 30]. The biomaterials not only play a role in physical support, protection and guidance, but their physicochemical properties, including degradation products, directly affect the microenvironment of peripheral nerve regeneration and have close bidirectional effects on surrounding tissues and cells. Our laboratory has been conducting a series of research works on peripheral nerve regeneration. We have taken the lead in developing porous chitosan artificial nerve grafts that are convenient for material exchange and vascular growth [31, 32]. The chitosan artificial nerve grafts have successfully bridged and repaired hundreds of cases of long-distance peripheral nerve defects [33], which obtained a Class III medical device registration certificate in 2020 in China (20203130898). The impact of biomaterials including chitosan conduits and inserted poly (lactic-co-glycolic acid) (PLGA) fibers on local regeneration microenvironment has been confirmed to be important [5].

After the nerve grafts are implanted into the body, chitosan is degraded into intermediate products, chitooligosaccharides (COS), by the action of tissue fluid and various enzymes, especially lysozyme. COS is a water-soluble substance with good biocompatibility, which has various biological activities, including anti-inflammatory, anti-tumor, antihypertensive and antioxidant [34]. Our research group illustrated that chitosan nerve grafts not only provide channels and scaffolds for peripheral nerve regeneration but also produce COS during the degradation process. As a neuroactive substance, COS can regulate the production of chemokines by SCs to induce macrophages to migrate to the injured site, regulate the nerve regeneration microenvironment and promote nerve regeneration [35]. Furthermore, COS is able to promote SC myelination and axon regeneration by regulating the secretion of extracellular vesicles from fibroblasts in the injured nerve [36, 37]. Then it is currently unknown whether COS has an impact on the regeneration preparation stage after peripheral nerve injury, especially on the clearance of tissue fragments especially myelin sheaths. The chitosan nerve graft in rat rapidly produce COS as it degrade, especially between 7 d and 14 d postoperatively [35, 38]. The clearance of tissue fragments mainly occurs in the early stage of nerve regeneration and may continue until about 4 weeks after surgery, when the proximal axon grows into the distal nerve trunk. This is necessary for us to fully understand the impact of chitosan and COS of artificial nerves on nerve regeneration, as well as the future design and application of other biomaterials in tissue engineered nerves. Our experiment observed the role of COS in myelin clearance during peripheral nerve regeneration, explored the cellular process of COS promoting myelin clearance and nerve regeneration and sought molecular intervention targets. This not only provides new theoretical support for the application of chitosan neural grafts and COS but also provides new methods and ideas for improving repair effects of artificial nerve grafts.

## Materials and methods

### Experimental animal

Adult male 8-week-old ICR mice were acquired from the Experimental Animal Center of Nantong University (License No. SYXK (Su) 2020–0029). The animals were housed in a temperature-controlled environment and allowed food and water *ad libitum*. The Administration Committee approved all experimental protocols of Experimental Animals, Jiangsu Province, China, following the guidelines of the Institutional Animal Care and Use Committee, Nantong University, China (No. P20230308-006).

### Animal model construction

The COS was prepared as previous [38, 39]. In brief, the chitosan refined was added in 15% H_2_O_2_ and irradiated with microwave power. The crude COS was re-dissolved in distilled water and purified. The eluted solution was lyophilized under vacuum. The purified COS has an average polymerization of approximately 7 with average molecular weight (MW) of approximately 900 Da. The degree of deacetylation was around 92.3%. Mice were deeply anesthetized through an intraperitoneal injection of 0.2 ml/10 g dose of 20 mg/ml tribromoethanol (Aibei, Nanjing, Jinagsu, China). The sciatic nerve at the left were exposed carefully. The experiments on effects of COS on nerve regeneration and myelin clearance involved two groups: the COS and normal saline (NS). The 4 μl of 0.12 mg/ml COS solution, or NS was injected perineurally using the disposable sterile insulin syringe (Becton, Dickinson and Company, Franklin Lake, NJ, USA). The sciatic nerve was crushed 2 mm for 30 s after the injection for 3 min, which was beneficial for the retention of COS injected in the nerve. In addition, the overexpression experiment *in vivo* was conducted to evaluate the role of WD repeat domain phosphoinositide-interacting protein 1 (Wipi1) mediating the COS effect. The 2 μl of AAV-Control or AAV-Wipi1 (OBiO, Shanghai, China) was injected perineurally before the crush injury. After the injection at 14 d, the autophagy inhibitor 3-methyladenine (3-MA) (MCE, NJ, USA) was given by intraperitoneal injection of 2 mg/1000 g once a day for a week. Then the crush injury of sciatic nerve was performed. Experiments of Wipi1 included four groups: the control, Wipi1, 3-MA and Wipi1 + 3-MA. After surgeries, the animals were kept in regular care. Then the evaluation of nerve regeneration was conducted at 14–28 days after surgery; the observation of myelin sheath clearance was conducted at 1–7 days or earlier time points.

### Rotarod test

The recovery of motor coordination was evaluated through mouse rota-rod treadmill (Muromachi Kikai, Tokyo, Japan). One week prior to the sciatic nerve injury, 30 min of daily pre training should be performed. Recorded the latency for mice to continuously rotate on a pole that was uniformly accelerated from 4 to 40 rpm/min at an acceleration of 20 rpm**/**min within 5 min. Each animal was tested at least 3 times, and the time interval between each test was greater than 5 min.

### Von Frey test

The recovery of mechanical pain was detected by Von Frey filaments pain meter (Danmic Global, San Jose, CA, USA). The middle part of the hind foot was slowly and gently stimulated with Von Frey filaments to observe the contraction reaction. The stimulating force started from small to large in sequence. The mechanical contraction threshold (MWT) was recorded. A time interval of at least 5 min between two stimuli was required.

### Plantar test

The recovery of thermal pain was detected by plantar thermal pain meter (IITC Life Science, Los Angeles, CA, USA) (Hargreaves method). The mice with a pain threshold of around 10–15 s were selected for the following experiment before surgeries. The thermal pain latency (TWL) from the thermal irradiation to the foot contraction was calculated. The irradiation interval was not less than 15 min, and each animal was measured 3 times. The maximum measurement time was 20 s to prevent damage to the feet.

### Catwalk analysis

The comprehensive recovery was estimated via Catwalk XT 10.6 (Noldus, Wageningen, Netherlands). The mice passed through a glass walkway, underneath which a video camera captured each run. The sciatic nerve function index (SFI) was calculated according to the formula [40].

### Electrophysiology analysis

Under deep anesthesia, the sciatic nerve was reexposed. Electrical stimuli with 5 mA and 2 Hz were applied to the distal and proximal ends of the crush segment sequentially. Compound muscle action potentials (CMAPs) were recorded on the gastrocnemius belly [41]. Nerve conduction velocities (NCVs) of the sciatic nerve were calculated. NCV = *d*/|*t*1-*t*2| (*t*1 is the proximal latency duration. *t*2 is the distal proximal latency duration. *d* is the distance between two electrode stimulation points).

### Tissue processing

The animals were anesthetized and perfused via the ascending aorta with NS and 4% paraformaldehyde (PFA). The distal regenerated nerves at a distance of 1 mm from crush sites were harvested. The collected nerves were then postfixed for 6–8 hr at 4°C, frozen, and cut into slices, respectively. The distal nerve tissues of crush sites used for ultrastructure observation were directly fixed in 2.5% glutaraldehyde (GA). In addition, the distal sciatic nerve samples for ribonucleic acid (RNA) and protein extractions were instantly collected and snap frozen in liquid nitrogen.

### Transmission electron microscope

The distal nerves of injury sites following fixation were collected and embedded in Epon 812 epoxy resin (Sigma). Ultrathin sections were obtained and stained with lead citrate and uranyl acetate. The photographs were obtained through a transmission electron microscope (TEM) (JEOL Ltd, Tokyo, Japan). The G-ratio and thickness of myelin of myelinated nerve fiber, and the myelinated nerve fiber (remak fiber) were calculated and analyzed. The autophagosomes were also observed and statistically analyzed.

### Immunofluorescence staining

The sections were blocked with 5% goat serum for 2 hr at room temperature, incubated with primary antibodies overnight at 4°C, and then, incubated with secondary antibodies for 1.5 hr at 37°C. Primary antibodies included rabbit anti-myelin basic protein (MBP) antibody (1:5000 dilution, Abcam), rabbit anti-S100 antibody (1:200 dilution, Abcam), mouse anti-S100 antibody (1:1000 dilution, Sigma), rabbit anti-lysosomal associated membrane protein 1 (LAMP1) antibody (1:100 dilution, Abcam), rabbit anti-microtubule-associated protein 1 light chain 3B (LC3B) antibody (1:1000 dilution, Abcam), mouse anti-p75 NGF receptor antibody (1:1000 dilution, Sigma), mouse anti-CD68 antibody (1:100 dilution, Abcam), rabbit anti-Wipi1 antibody (1:50 dilution, Abcam) and mouse anti-NF200 antibody (1:200 dilution, Sigma). Secondary antibodies included goat anti-rabbit IgG-Alex-647 (1:500 dilution, Abcam), goat anti-rabbit IgG-Alex-488 (1:400 dilution, Abcam) and goat anti-mouse IgG Cy3 (1:1000 dilution, Abcam). Nuclei were marked using DAPI staining solution (Beyotime, Shanghai, China). Images were acquired under fluorescence microscopy (Carl Zeiss, Oberkochen, Baden-Würburg, Germany). Quantitative analyses including area and intensity were performed via ImageJ (https://imagej.nih.gov/ij/index.html).

### Fluorescent myelin staining

The FluoroMyelin Red Fluorescent Myelin Stain (ThermoFisher) was used. Prepare the staining solution by diluting the stock solution 300-fold into phosphate buffer saline (PBS). Flood the sciatic nerve sections with staining solution for 20 min at room temperature. When staining is complete, wash 3 times for 10 min each. Images were acquired under fluorescence microscopy (Zeiss). Quantitative analyses including area and intensity were performed via ImageJ.

### Myelin oil red staining

The sciatic nerve sections were stained using Modified Oil Red O (ORO) Staining Kit (Beyotime Biotechnology). Added an appropriate amount of washing solution to cover the sample for 20 s. Immersed the slices in staining solution and stained for 30 min. The Slices were washed on a shaker for 20 s in distilled water following the washing solution addition for 30 s. Nuclei were restained using hematoxylin (Beyotime Biotechnology). Images were acquired under fluorescence microscopy in brightfield (Zeiss). Quantitative analyses were performed via ImageJ.

### Myelin chromotrope staining

The Myelin Stain Kit (Chromotrope 2R Method) (Solarbio Life Sciences) was used. The nerve slices were stained for 15 min in Chromotrope 2R solution, and washed directly for 2–3 times in 2R differentiation solution. Then the staining with light green solution for 10 min was conducted. Conventionally dehydrate, transparent and seal. The myelin sheaths presented dark red. The axons and stromas presented green. Images were acquired under fluorescence microscopy in brightfield (Zeiss). Quantitative analyses were performed via ImageJ.

### Real-time polymerase chain reaction

Total RNA of the sciatic nerve was extracted. Reverse-transcribed complementary deoxyribonucleic acid (DNA) was synthesized with the Prime-Script RT Reagent Kit (TaKaRa, Dalian, Shandong, China). qPCR was performed with SYBR Premix Ex Taq (TaKaRa, Dalian, Shandong, China). The sequences of primer pairs are as follows: 18SrRNA (5’-3’) forward, TCTATTTTGTTGGTTTTCGG; reverse, ATGCTTTCGCTCTGGTTC. Wipi1 (5’-3’) forward, CTGCTTCTCTTTCAACCAAGACT; reverse, ACGTCAGGGATTTCATTGCTT. Bloc1s2 (5’-3’) forward, AGCCACCAGTGAAGACTACAA; reverse, CTGCTCCTCAATCATGTTGATCT. NPC1 (5’-3’) forward, TGTTTGGTATGGAGAGTGTGGA; reverse, GTCACAGCAGAGACTGACATTG. Stx5 (5’-3’) forward, CGTTTCCTCCTGTTCTTTA; reverse, ATTGACATGATGGACCCTA. Lamtor1 (5’-3’) forward, TAGCAGCGAAAACGAGGACTC; reverse, TGAAGGTAGGCTATGGTAGTTGG. TSPO (5’-3’) forward, CGTCCTCTGTGAAACCTCCC; reverse, AAACCCTCTTGGCATCCG. The data obtained from three independent experiments.

### Western blot

The sciatic nerves were homogenized in RIPA lysis buffer, thoroughly ground and further centrifuged at 12 000 g at 4°C for 20 min. The protein concentrations were detected using BCA Protein Assay Kit (Beyotime). Equal amount proteins were loaded onto 7.5–12.5% SDS-PAGE gel, electrophoresed and transferred to polyvinylidene fluoride (PVDF) membranes. The PVDF membrane was blocked with 5% non-fat milk at room temperature for 2 hr, incubated with primary antibodies overnight at 4°C and appropriate secondary antibodies for 1 hr at room temperature. Primary antibodies involved anti-MBP antibody (1:1000 dilution, Abcam), anti-Wipi1 antibody (1:2000 dilution, Abcam), anti-myelin protein zero (MPZ) antibody (1:1000 dilution, Abcam) and anti-β-actin antibody (1:4000 dilution, Abcam). Immunoblots were visualized with High-sig ECL Western Blotting substrate (Tanon, Guangzhou, Guangdong, China) and quantified using the ImageJ software (https://imagej.nih.gov/ij/index.html).

### Proteomics and bioinformatics analysis

The 4D-Label free proteomics analysis of the sciatic nerve samples in different groups was performed. Briefly, an appropriate amount of protein from each sample was taken for trypsin enzymatic hydrolysis using the filter aided sample preparation (FASP) method. After chromatographic separation, the sample was subjected to mass spectrometry analysis using the timsTOF Pro mass spectrometer. LC-MS/MS analysis was conducted, and the resulting MS/MS data were processed using the MaxQuant search engine (v.1.6.14). Proteins with an absolute fold change (FC) > 1.5 and *P* values < 0.05 were considered as differential expressions. Then the Gene Ontology (GO) by Blast2 GO (https://www.blast2go.com/) and Kyoto Encyclopedia of Genes and Genomes (KEGG) by KEGG Automatic Annotation Server (KAAS) (https://www.genome.jp/kegg/kaas/) annotation and enrichment analysis were performed for the target protein set.

### Triglyceride and total cholesterol measurement

Contents of triglyceride (**TG**) and total cholesterol (THO) were determined using Triglyceride Colorimetric Assay Kit (Nanjing Jiancheng, A110-1-1) and THO assay kit (Nanjing Jiancheng, A111-1-1), respectively. The sciatic nerves of four groups were extracted. The weight of tissue was accurately weighed, and 9 times the volume of NS was added according to the ratio of weight (g): volume (ml) = 1: 9, homogenized under ice water bath conditions and centrifuged at 2500 rpm for 10 min. The 500 μl tissue homogenate was determined using a TG assay kit. THO detection method is the same as above.

### Structure-based protein interaction interface analysis

The interactome network was further analyzed using the STRING website (https://cn.string-db.org/). The significance of network edges (‘confidence’), the active interaction sources (‘experiments’), the maximum number of participants to display (‘no more than 50 interactors’) and the minimal interaction score [‘Medium confidence (0.400)’] were selected to reduce the bias. Structures of Wipi1, Atg2a and Atg2b were downloaded from the PDB database. The potential interface prediction of Wipi1 with Atg2a and Atg2b was obtained by PRISM tool (https://cosbi.ku.edu.tr/prism). Prediction results were visualized using the PyMol tool (https://pymol.org).

### Statistical analysis

The data were presented as means ± standard deviation (SD). *T*-test was used between two groups. One-way analysis of variance (one-way ANOVA) was used for multiple group comparisons. Two-way analysis of variance (two-way ANOVA) was used for multiple group comparisons at different time points. Graph-Pad Prism 8.0 software (GraphPad Software Inc., La Jolla, CA, USA) was used. A *P* values < 0.05 was considered significant statistically.

## Results

### Chitooligosaccharides are beneficial for peripheral nerve regeneration

**Figure 1.**
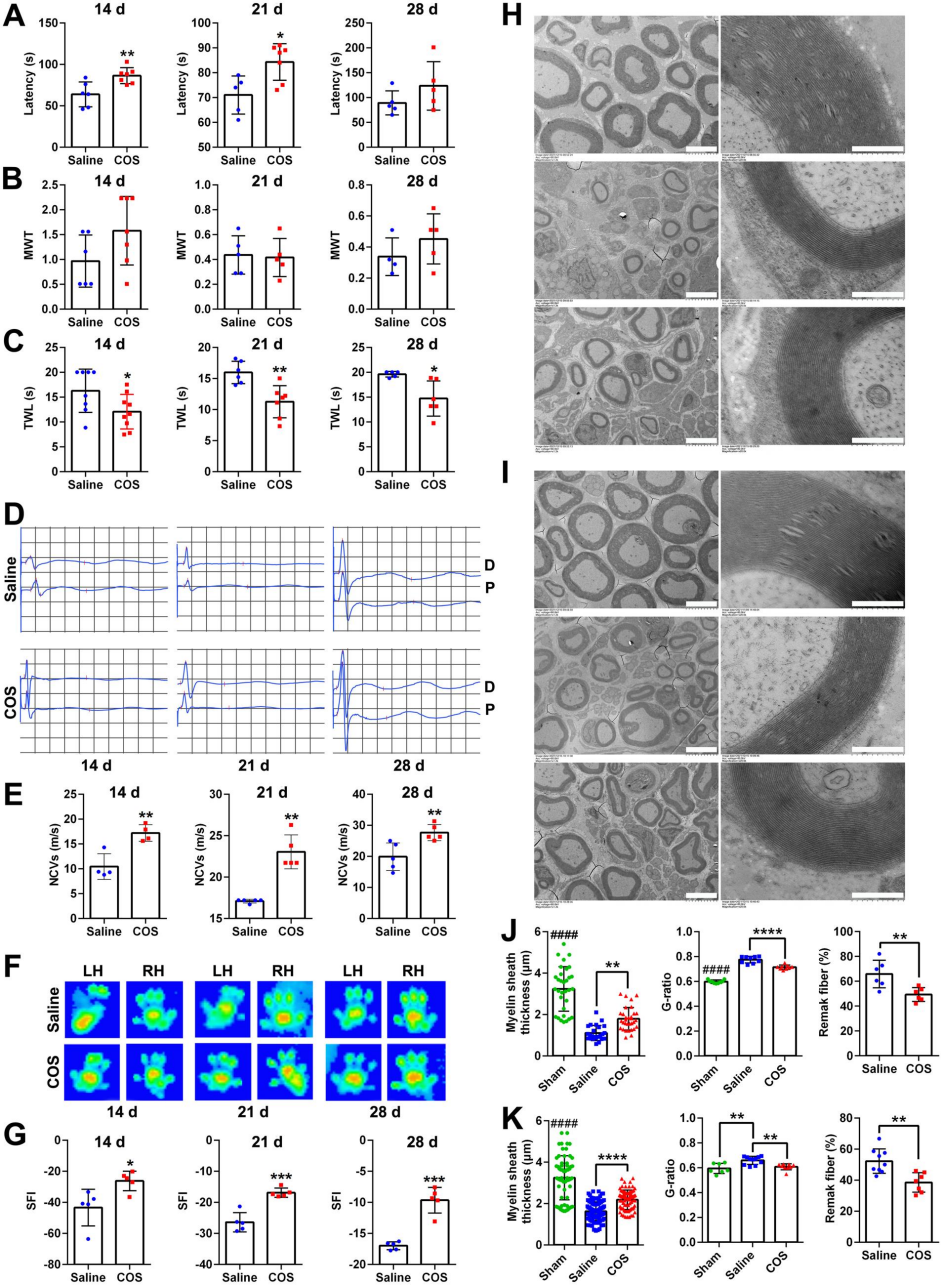
The promoting effect of chitooligosaccharides on nerve regeneration. (**A**) Histograms of the latency of rotarod test at 14 d, 21 d and 28 d after injuries, respectively, *n* ≥ 5. The latency of COS group was significantly higher. *, *P* < 0.05; **, *P* < 0.01. (**B**) Histograms of the MWT of Von Frey test at 14 d, 21 d and 28 d after injuries, respectively, *n* ≥ 4. (**C**) Histograms of the TWL of plantar test at 14 d, 21 d and 28 d after injuries, respectively, *n* ≥ 5. The TWL of COS group was significantly lower. *, *P* < 0.05; **, *P* < 0.01. (**D**) Representative waveforms of the electrophysiology at 14 d, 21 d and 28 d after injuries. *D* indicated the distal recording point of the crush segment. *P* indicated the proximal recording point of the crush segment. (**E**) Histograms of the NCVs at 14 d, 21 d and 28 d after injuries, respectively, *n* ≥ 4. The NCVs of COS group was significantly higher. **, *P* < 0.01. **(F**) Representative footprints at 14 d, 21 d and 28 d after injuries. LH indicated the surgical side on left. RH indicated the healthy side on right. (**G**) Histograms of the SFI at 14 d, 21 d and 28 d after injuries, respectively, *n* = 5. The SFI of COS group was significantly higher. *, *P* < 0.05; ***, *P* < 0.001. (**H** and **I**) Representative ultrastructural images of the sham, saline and COS groups at 14 d (**H**) and 28 d (**I**) after injuries, respectively. The images on right showed an enlarged local field of myelin sheath layers on left. Scale bar, 5 μm and 500 nm, respectively. (**J**) Histograms of the myelin sheath thickness, *G*-ratio and percentage of remak fiber at 14 d after injuries, *n* ≥ 6. *, each group vs. saline group. #, each group vs. sham group. **, *P* < 0.01; ****, *P* < 0.0001. ####, *P* < 0.0001. (**K**) Histograms of the myelin sheath thickness, *G*-ratio and percentage of remak fiber at 28 d after injuries, *n* ≥ 7. *, each group vs. saline group. #, each group vs. sham group. **, *P* < 0.01; ****, *P* < 0.0001. ####, *P* < 0.0001.

The nerve function recovery tests were performed at 14 d, 21 d and 28 d post injury. The latency time on the rotating stick of the COS group was significantly longer than that of the saline group (Figure 1A), indicating the better recovery of motor coordination and balance abilities. In terms of the mechanical pain recovery, The MWT showed no significant difference (Figure 1B). Meanwhile the TWL of the COS group was significantly shortened (Figure 1C), indicating the better recovery of thermal pain. Then the neurophysiological tests showed that the COS group had the better recovery in both NCVs and CMAPs (Figure 1D and E). Further overall functional gait evaluation demonstrated a significant improvement in SFI of the COS group (Figure 1F and G). Through the ultrastructural observation of the distal regenerated nerve to present the sizes of myelins and axons, especially the layers of myelin sheaths at 14 d and 28 d postoperation (Figure 1H and I), the myelin sheath regeneration of the COS group, including myelin thickness, G-ratio and proportion of myelinated nerve fibers, were also significantly better than saline group (Figure 1J and K).

### Chitooligosaccharides accelerate myelin clearance

**Figure 2.**
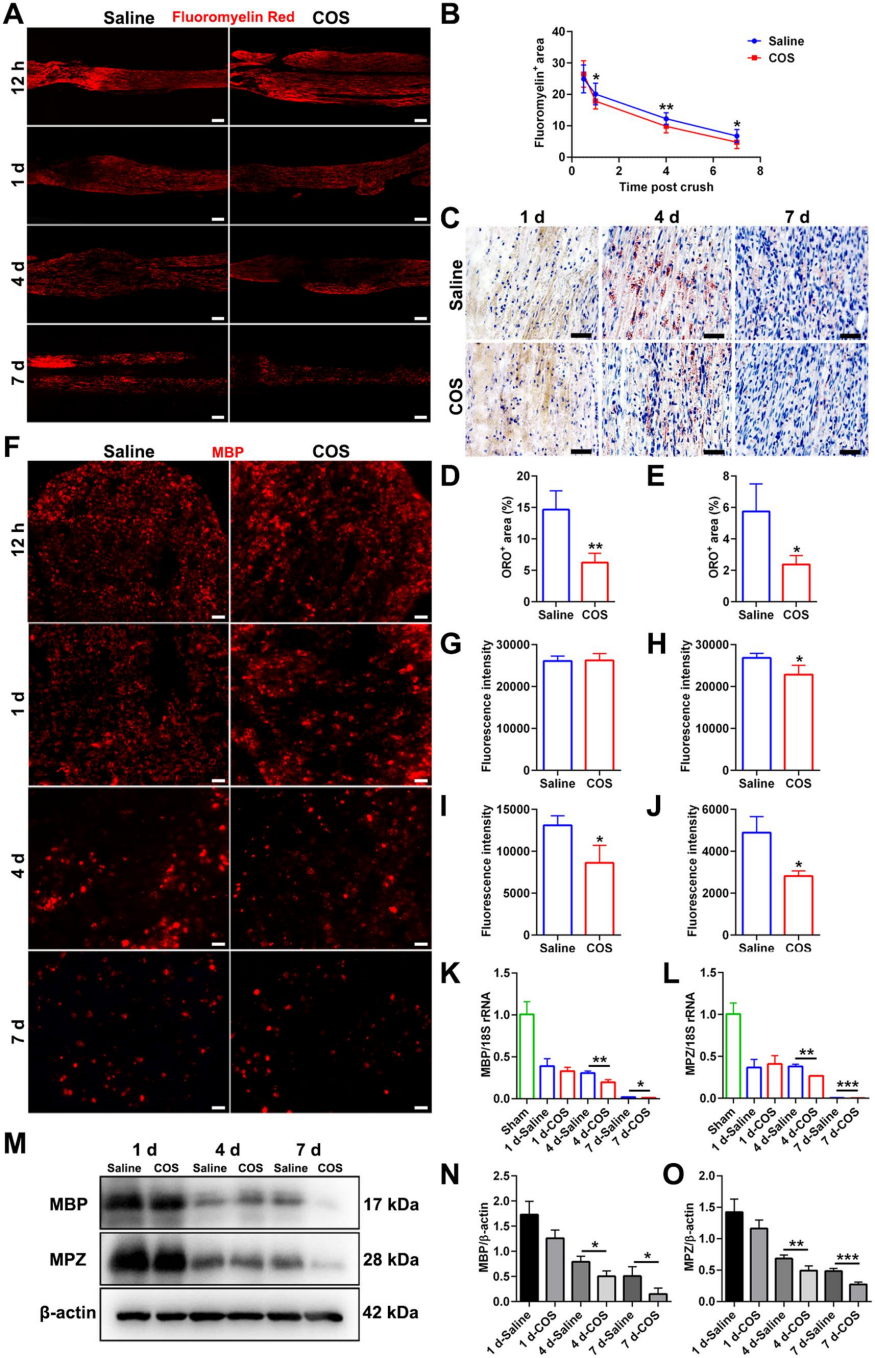
The promoting effect of chitooligosaccharides on myelin clearance after peripheral nerve injury. (**A**) Representative images of the fluoromyelin red staining at 12 hr, 1 d, 4 d and 7 d after injuries. Scale bar, 200 μm. (**B**) Line chart of the positive fluoromyelin area post crush. The area of COS group was significantly lower at 1 d, 4 d and 7 d, *n* = 3. *, *P* < 0.05; **, *P* < 0.01. (**C**) Representative images of the oil red O staining at 1 d, 4 d and 7 d after injuries. The lipid droplets appeared red. Scale bar, 20 μm. (**D, E**) Histograms of the percentage of positive oil red O area at 4 d and 7 d after injuries, respectively, *n* = 3. *, *P* < 0.05; **, *P* < 0.01. (**F**) Representative images of the MBP staining at 12 hr, 1 d, 4 d and 7 d after injuries. Scale bar, 20 μm. (**G**–**J**) Histograms of the MBP fluorescence intensity at 12 hr, 1 d, 4 d and 7 d after injuries, respectively, *n* = 3. *, *P* < 0.05; **, *P* < 0.01. (**K, L**) Histogram of the polymerase chain reaction (PCR) of MBP and MPZ at 1 d, 4 d and 7 d after injuries, respectively, *n* = 3. The differences between two groups were statistically significant at 4 d and 7 d. *, *P* < 0.05; **, *P* < 0.01; ***, *P* < 0.001. (**M**) The images of the expressions of MBP and MPZ proteins at 1 d, 4 d and 7 d after injuries. (**N, O**) Histogram of the Western blot of MBP and MPZ at 1 d, 4 d and 7 d after injuries, respectively, *n* = 3. The differences between the two groups were statistically significant at 4 d and 7 d. *, *P* < 0.05; **, *P* < 0.01; ***, *P* < 0.001.

The effect of COS on myelin debris clearance after nerve injury was observed through staining of myelin sheath lipids. The distal nerves were stained using myelin lipid specific dye FluoroMyelin Red. As the time after nerve injury prolonged, the myelin signals gradually decreased in both groups (Figure 2A). At 12 h, there was no significant difference. But the fluorescence signals of COS group were significantly reduced at 1 d, 4 d and 7 d, which were lower than those of saline group with statistical significances (Figure 2B). Meanwhile, the staining of lipid droplets derived from the degradation of myelin lipids was conducted using ORO. There were no ORO staining signals in the two groups at 1 d. Then, at 4 d and 7 d, the accumulation of lipid droplets from myelin degradation in both groups were revealed, while the COS treatment significantly reduced lipid droplet accumulation (Figure 2C–E). However, the lipid droplets in myelin sheath were no longer obvious at 7 d. In addition, the MBP immunofluorescence staining displayed that the cross-section of myelin surrounding axon was circular, and the ring structure of myelin gradually decreased. The reduction of MBP fluorescence intensity of the COS group at 1 d, 4 d and 7 d were significantly faster (Figure 2F–J). Furthermore, the myelin related molecular detections with significant differences in morphological myelin clearance between two groups were conducted. At 1 d after injury, there was no significant difference in the MBP and MPZ expressions. At 4 d and 7 d after injury, the gene expressions of MPZ and MBP of COS group were significantly lower (Figure 2K and L). The protein expressions of MBP and MPZ of COS group were significantly lower at 4 d and 7 d too (Figure 2M–O).

### Chitooligosaccharides enhance Schwann cell autophagy

**Figure 3.**
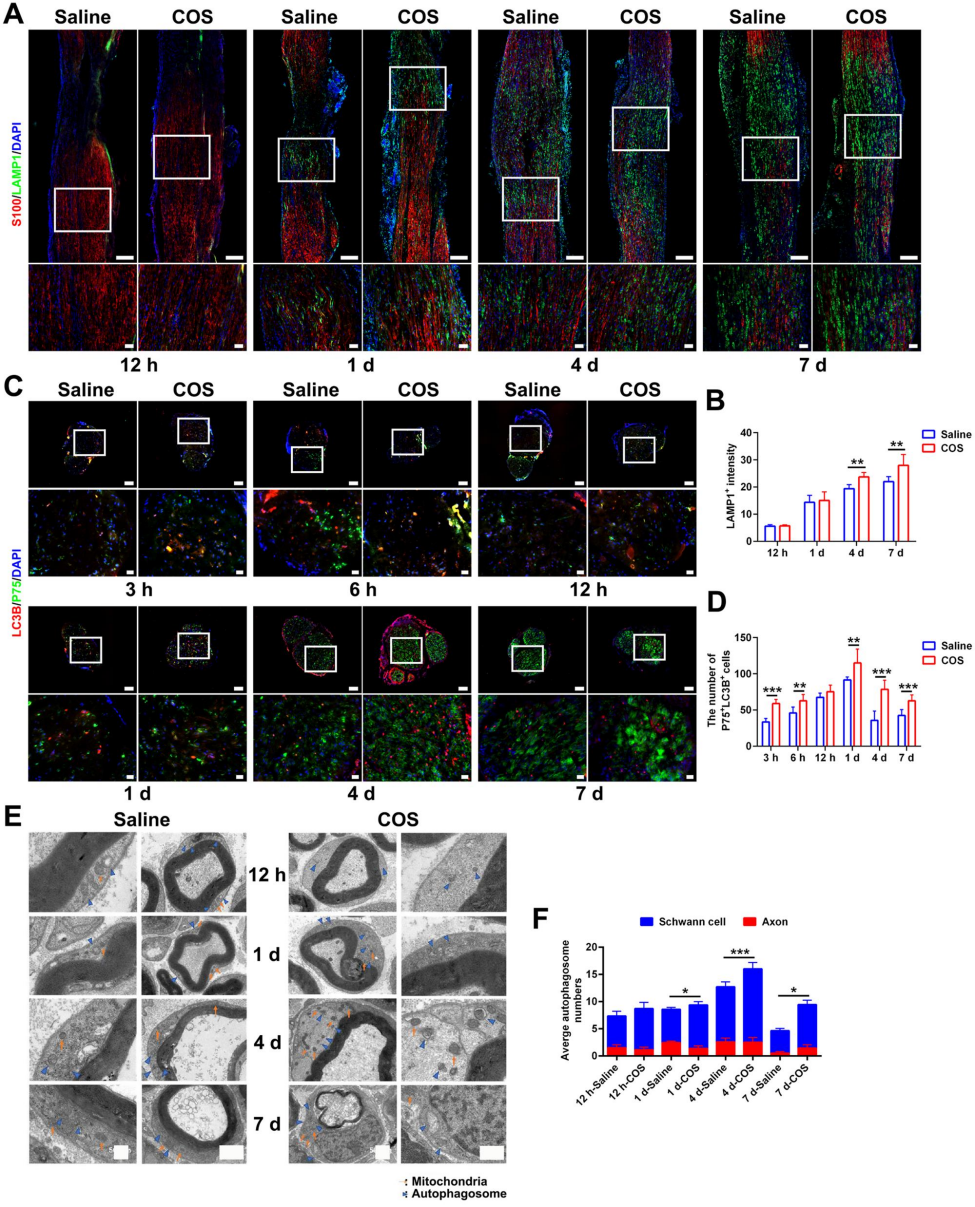
The effect of chitooligosaccharides on Schwann cell autophagy after peripheral nerve injury. (**A**) Representative immunofluorescence images of the Schwann cell lysosomes at 12 hr, 1 d, 4 d and 7 d after injuries, respectively. The images below displayed an enlarged field of the box. The S100 labelled Schwann cells. The LAMP1 labelled lysosomes. The DAPI labelled nuclei. Scale bar, 200 μm and 50 μm, respectively. (**B**) Histograms of the LAMP1 fluorescence intensity at 12 hr, 1 d, 4 d and 7 d after injuries, respectively, *n* = 3. The differences between two groups were statistically significant at 4 d and 7 d. **, *P* < 0.01. (**C**) Representative immunofluorescence images of the Schwann cell autophagosomes at 3 hr, 6 hr, 12 hr, 1 d, 4 d and 7 d after injuries, respectively. The images below displayed an enlarged field of the box. The LC3B labelled autophagosomes. The P75 labelled Schwann cells. The DAPI labelled nuclei. Scale bar, 100 μm and 20 μm, respectively. (**D**) Histograms of the number of double labelled cells at 3 hr, 6 hr, 12 hr, 1 d, 4 d and 7 d after injuries, respectively, *n* = 3. The differences between two groups were statistically significant at 3 hr, 6 hr, 1 d, 4 d and 7 d. **, *P* < 0.01; ***, *P* < 0.001. (**E**) Representative ultrastructural images of the Schwann cell autophagosomes at 12 hr, 1 d, 4 d and 7 d after injuries, respectively. Arrows indicated mitochondria. Tailless arrows indicated autophagosomes. Scale bar, 2 μm and 500 nm, respectively. (**F**) Histograms of the number of Schwann cell autophagosomes at 12 hr, 1 d, 4 d and 7 d after injuries, respectively, *n* = 3. The differences between two groups were statistically significant at 1 d, 4 d and 7 d. *, *P* < 0.05; ***, *P* < 0.001.

**Figure 4.**
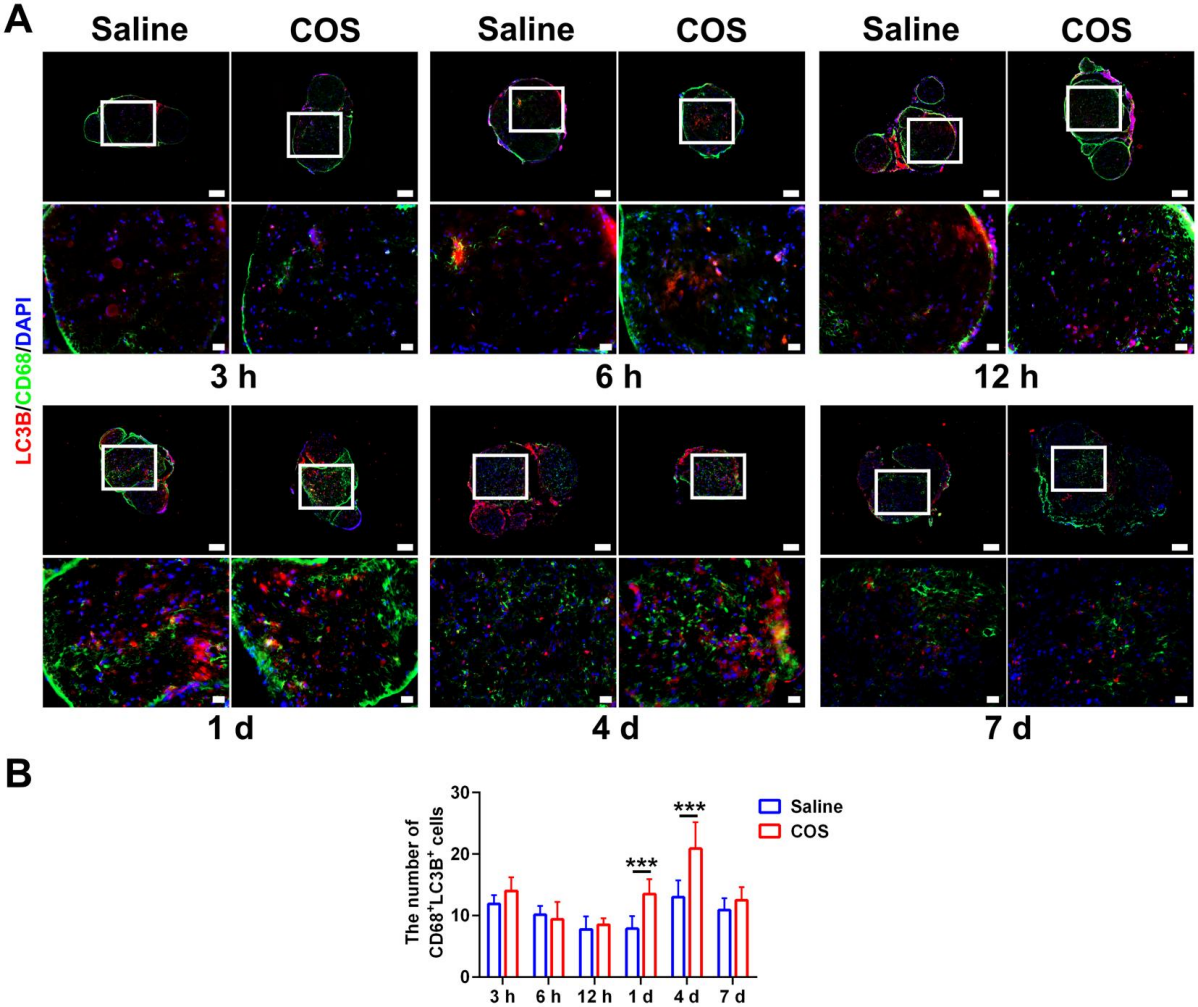
The effect of chitooligosaccharides on macrophage autophagy after peripheral nerve injury. (**A**) Representative immunofluorescence images of the macrophage autophagosomes at 3 hr, 6 hr, 12 hr, 1 d, 4 d and 7 d after injuries, respectively. The images below displayed an enlarged field of the box. The LC3B labelled autophagosomes. The CD68 labelled macrophages. The DAPI labelled nuclei. Scale bar, 100 μm and 20 μm, respectively. (**B**) Histograms of the number of double labelled cells at 3 hr, 6 hr, 12 hr, 1 d, 4 d and 7 d after injuries, respectively, *n* = 3. The differences between two groups were statistically significant at 1 d and 4 d. ***, *P* < 0.001.

The clearance of myelin in later stage is closely related to the lysosomes and autophagy. The experiment analyzed the relationship among lysosomes, autophagosome and SCs. As the injury time prolonged, accumulation of LAMP1 could be seen in the myelin lysis area at the distal nerve, and it was colocalized with SCs (Figure 3A). At 4 d and 7 d after injury, the fluorescence intensity of LAMP1 of COS group further increased, indicating an increase in myelin clearance activity (Figure 3B). Then the LC3B staining illustrated that the number of autophagosomes in SCs (P75^+^LC3B^+^) of two groups gradually increased and decreased at 4 d and 7 d (Figure 3C). The number of colabeled autophagosomes of the COS group was significantly higher (Figure 3D). Moreover, under the electron microscope, typical structures of autophagosomes could be observed at 12 h and 1 d, presenting a double-layer or multi-layer vacuolar structure; and the isolation membrane contained various cytoplasmic components that needed to be degraded, such as mitochondria, ribosomes and endoplasmic reticulum. At 4 d, plurality autophagosomes and lysosomes fused to form a monolayer membrane structure of autophagosomes; and the cytoplasmic components inside the isolation membrane showed degradation, accompanied by the appearance of a large number of mitochondria exhibiting a layered cristae structure with the swelling, degeneration and other phenomena (Figure 3E). The changes in the number of autophagosomes at 12 h and 1 d were not yet significant. At 4 d, the number of autophagosomes increased significantly compared to 12 h, and decreased slightly at 7 d. The number of autophagosomes in SCs of COS group were significantly higher at 1 d, 4 d and 7 d (Figure 3F). Yet the colabeling staining on macrophages and autophagosomes showed that an increasing colocalization trend was seen only at 1 d and 4 d, and there was a statistical difference between two groups (Figure 4A and B).

### Chitooligosaccharides regulate Schwann cell autophagy through Wipi1 mediation

**Figure 5.**
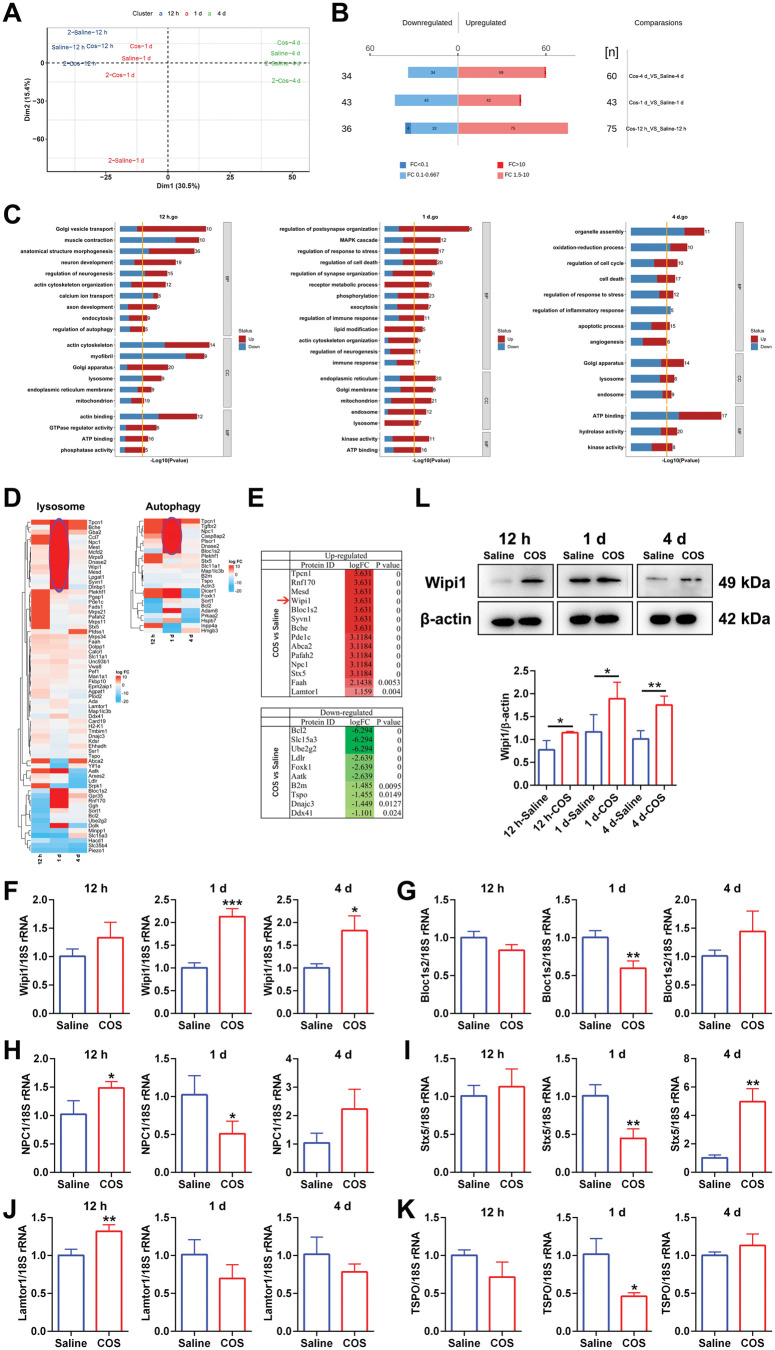
Proteomics, bioinformatics analysis and expression verification. (**A**) Image of the PCA dimensionality reduction data processing. (**B**) Image of the differentially expressed proteins including upregulated proteins and downregulated proteins at 12 hr, 1 d and 4 d after injuries. (**C**) The GO enrichment analysis of differentially expressed proteins at 12 hr, 1 d and 4 d after injuries, respectively. The biological process (BP), cellular component (CC) and molecular function (MF) were involved. The ‘regulation of autophagy’ of BP was enriched at 12 hr. The ‘lysosome’ of CC was enriched at three time points. (**D**) Heatmap of the proteins enriched in lysosome and autophagy related regulations. The ellipse indicated a significant upregulation at 1 d after injury, and other time points also mainly showed upregulated molecular sets. (**E**) Top differentially expressed proteins including upregulated and downregulated proteins comprehensively screened regardless of time points according to both the log FC and *P* values. Arrow indicated Wipi1, which was the most significantly upregulated proteins regulating autophagy directly and was selected for in-depth validation. (**F**–**K**) PCR expression validations of differentially expressed molecules at 12 hr, 1 d and 4 d after injuries, respectively. The Wipi1 was significantly upregulated. *, *P* < 0.05; **, *P* < 0.01; ***, *P* < 0.001. (**L**) Western blot validations of Wipi1 at 12 hr, 1 d and 4 d after injuries. The differences between the two groups were all statistically significant. *, *P* < 0.05; **, *P* < 0.01.

**Figure 6.**
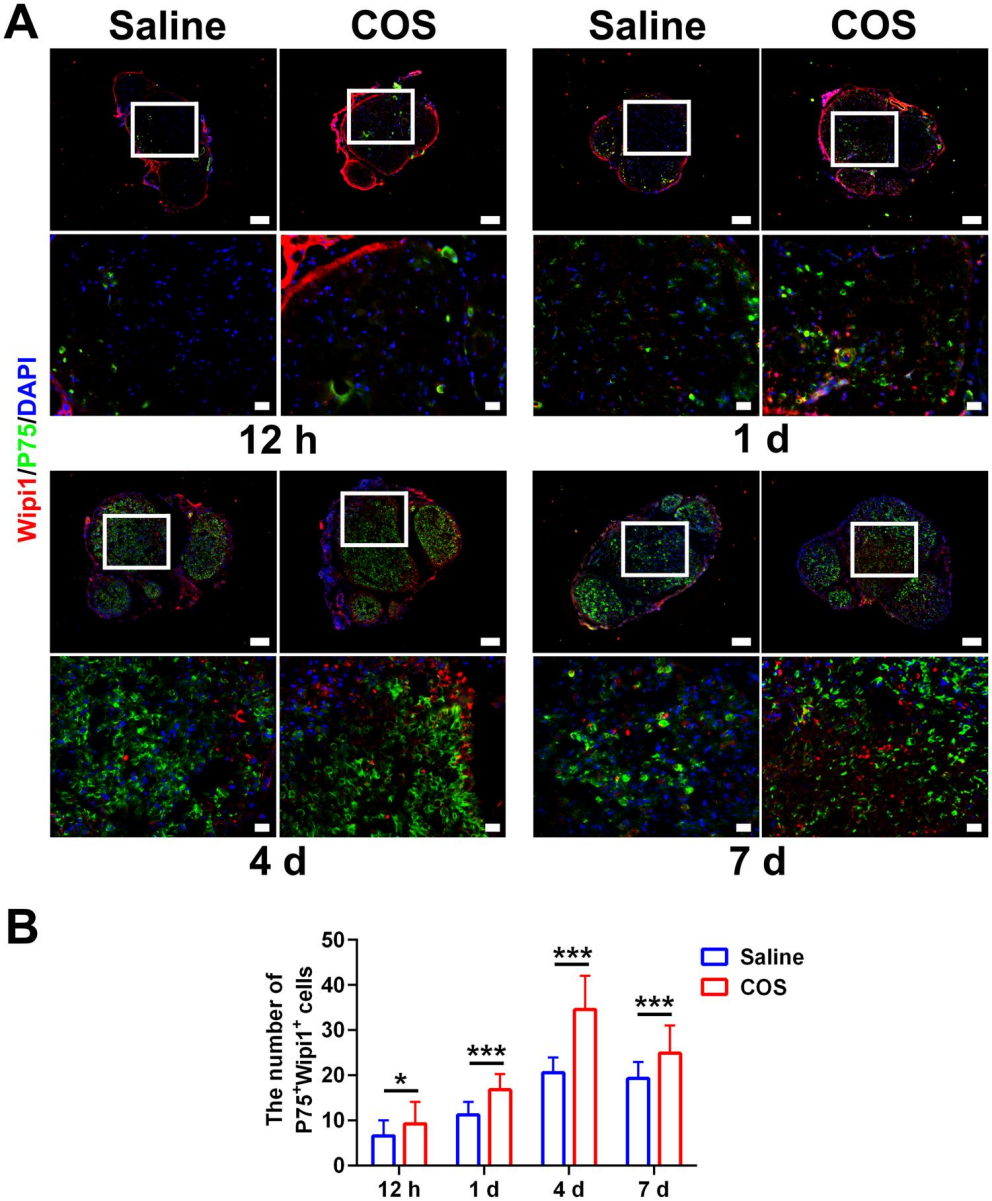
The effect of chitooligosaccharides on Wipi1 expression after peripheral nerve injury. (**A**) Representative immunofluorescence images of the Wipi1 expression in Schwann cells at 12 hr, 1 d, 4 d and 7 d after injuries, respectively. The images below displayed an enlarged field of the box. The P75 labelled Schwann cells. The DAPI labelled nuclei. Scale bar, 100 μm and 20 μm, respectively. (**B**) Histograms of the number of double labelled cells at 12 hr, 1 d, 4 d and 7 d after injuries, respectively, *n* = 3. The differences between the two groups were all statistically significant. *, *P* < 0.05; ***, *P* < 0.001.

The distal nerve samples at 12 h, 1 d and 4 d were subjected to proteomic analysis. Principal component analysis (PCA) demonstrated that the visible homogeneity of protein expression between repeated samples of both groups (Figure 5A). The COS group had 111, 86 and 94 differentially expressed proteins, respectively, at three time points compared to the saline group (Figure 5B) (Supporting Information Table S1). GO analysis found that differential proteins were closely related to lysosomal function, autophagy regulation, regulation of neurogenesis and cytoskeleton (Figure 5C). Meanwhile KEGG pathway annotation displayed that some of autophagic active pathways were involved in this process of COS treatment (Supporting Information Figure S1). The heatmaps of proteins related to lysosomes and autophagy containing duplicate sample values at different time points were separately painted too (Supporting Information Figure S2). The differentially expressed proteins involved in lysosomes and autophagy at different time points were further integrated into heatmaps (Figure 5D), of which the proteins with the highest differential expression amplitude and the maximum reliability comprehensively were screened. Wipi1 was the most direct and classic regulatory factor related to autophagy in top five significantly upregulated proteins (Figure 5E). The gene transcription levels of some differential proteins at 12 h, 1 d and 4 d, respectively, were validated through qPCR (Figure 5F–K). The expression of Wipi1 gene had been consistently increasing, especially at 1 d and 4 d after injury, which was consistent with proteomic results (Figure 5F). Wipi1 protein expression of COS group was significantly increased at each time point and consistent with the qPCR and proteomics (Figure 5L). The further immunohistochemical stainings were performed on SCs and Wipi1, which was expressed in the cytoplasm of SCs (Figure 6A). The number of autophagosomes in SCs gradually increased from 12 h, 1 d to 7 d followed by a slight decrease, which of COS group was significantly higher at each time point (Figure 6B).

### Wipi1 promotes the recovery of peripheral nerve structure and function

**Figure 7.**
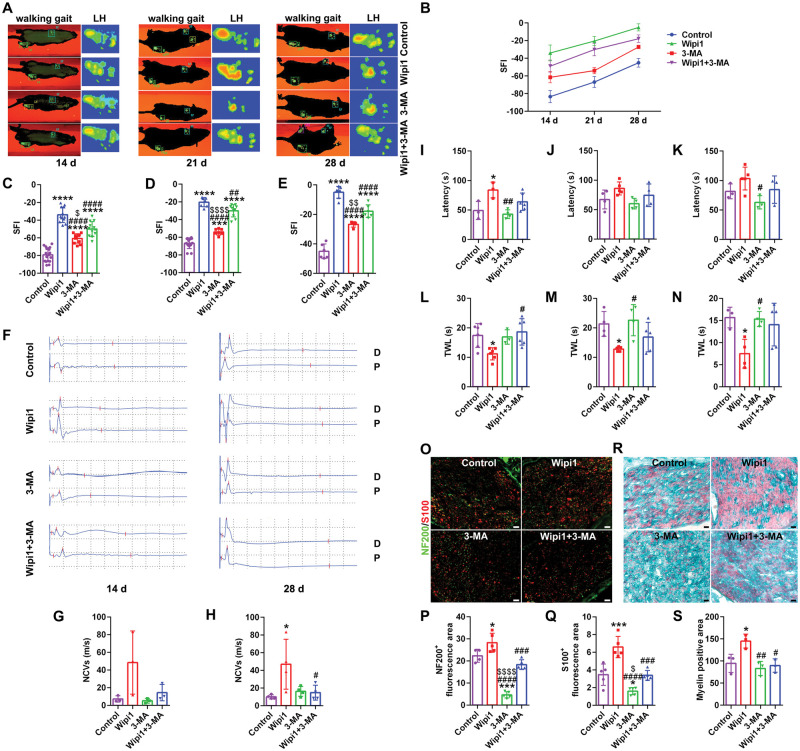
The promoting effect of Wipi1 on peripheral nerve regeneration. (**A**) Representative footprints at 14 d, 21 d and 28 d after injuries. LH indicated the surgical side on left. (**B**) Dynamic line chart of SFI. The SFI recoveries of Wipi1 group were faster at all time points. (**C**–**E**) Histograms of the SFI at 14 d, 21 d and 28 d after injuries, respectively, *n* ≥ 5. *, each group vs. control group. #, each group vs. Wipi1 group. $, each group vs. Wipi1 + 3-MA group. ****, *P* < 0.0001. ##, *P* < 0.01; ####, *P* < 0.0001. $, *P* < 0.05; $$, *P* < 0.01; $$$$, *P* < 0.0001. (**F**) Representative waveforms of the electrophysiology at 14 d and 28 d after injuries. D indicated the distal recording point of the crush segment. *P* indicated the proximal recording point of the crush segment. (**G, H**) Histograms of the NCVs at 14 d, 21 d and 28 d after injuries, respectively, *n* ≥ 3. *, each group vs. control group. #, each group vs. Wipi1 group. *, *P* < 0.05. #, *P* < 0.05. (**I**–**K**) Histograms of the latency of rotarod test at 14 d, 21 d and 28 d after injuries, respectively, *n* ≥ 3. *, each group vs. control group. #, each group vs. Wipi1 group. *, *P* < 0.05. #, *P* < 0.05; ##, *P* < 0.01. (**L**–**N**) Histograms of the TWL of plantar test at 14 d, 21 d and 28 d after injuries, respectively, *n* ≥ 3. *, each group vs. control group. #, each group vs. Wipi1 group. *, *P* < 0.05. #, *P* < 0.05. (**O**) Representative immunofluorescence images of the dital regenerated nerves at 28 d after injury. The S100 labelled Schwann cells. The NF200 labelled axons. The DAPI labelled nuclei. Scale bar, 20 μm. (**P, Q**) Histograms of the positive NF200 area and S100 area at 28 d after injury, respectively, *n* ≥ 4. *, each group vs. control group. #, each group vs. Wipi1 group. $, each group vs. Wipi1 + 3-MA group. *, *P* < 0.05; ***, *P* < 0.001. ###, *P* < 0.001; ####, *P* < 0.0001. $, *P* < 0.05; $$$$, *P* < 0.0001. (**R**) Representative myelin chromotrope staining images of the dital regenerated nerves at 28 d after injury. The red labelled myelin. Scale bar, 20 μm. (**S**) Histogram of the positive myelin area at 28 d after injury, *n* = 3. *, each group vs. control group. #, each group vs. Wipi1 group. *, *P* < 0.05. #, *P* < 0.05; ##, *P* < 0.01.

Effects of Wipi1 protein on promoting nerve regeneration were evaluated. The catwalk analysis showed that the overall functional recoveries of Wipi1 group were relatively faster and better, and both the soles and toes were able to more effectively support the walking body under nerve reinnervation (Figure 7A). The calculation and analysis of SFI displayed that at all time points, the Wipi1 group had significantly better recoveries; and the inhibitor 3-MA could significantly prevent the recovery of SFI, although the differences were also statistically significant (Figure 7A–E). The electrophysiological detection also demonstrated a similar trend, that was, Wipi1 significantly promoted the effective transmission of regenerative nerve electrical signals (Figure 7F–H). In addition, the latency of rotarod test were significantly longer than the control group at 14 d, and slightly longer at 21 d and 28 d without statistical significance (Figure 7I–K). The TWL of plantar test of the Wipi1 group were significantly shortened at all-time points (Figure 7L–N). Furthermore, the regeneration of crushed nerves was stained and observed. The fluorescence signal areas of regenerated axons and SCs of the Wipi1 group were significantly larger than the control group (Figure 7O–Q). The myelin sheath area of the Wipi1 group was higher with the better recovery of tissue structure (Figure 7R and S).

### Wipi1 interacts with the Atg2 to mediate myelin clearance and lipid metabolism

**Figure 8.**
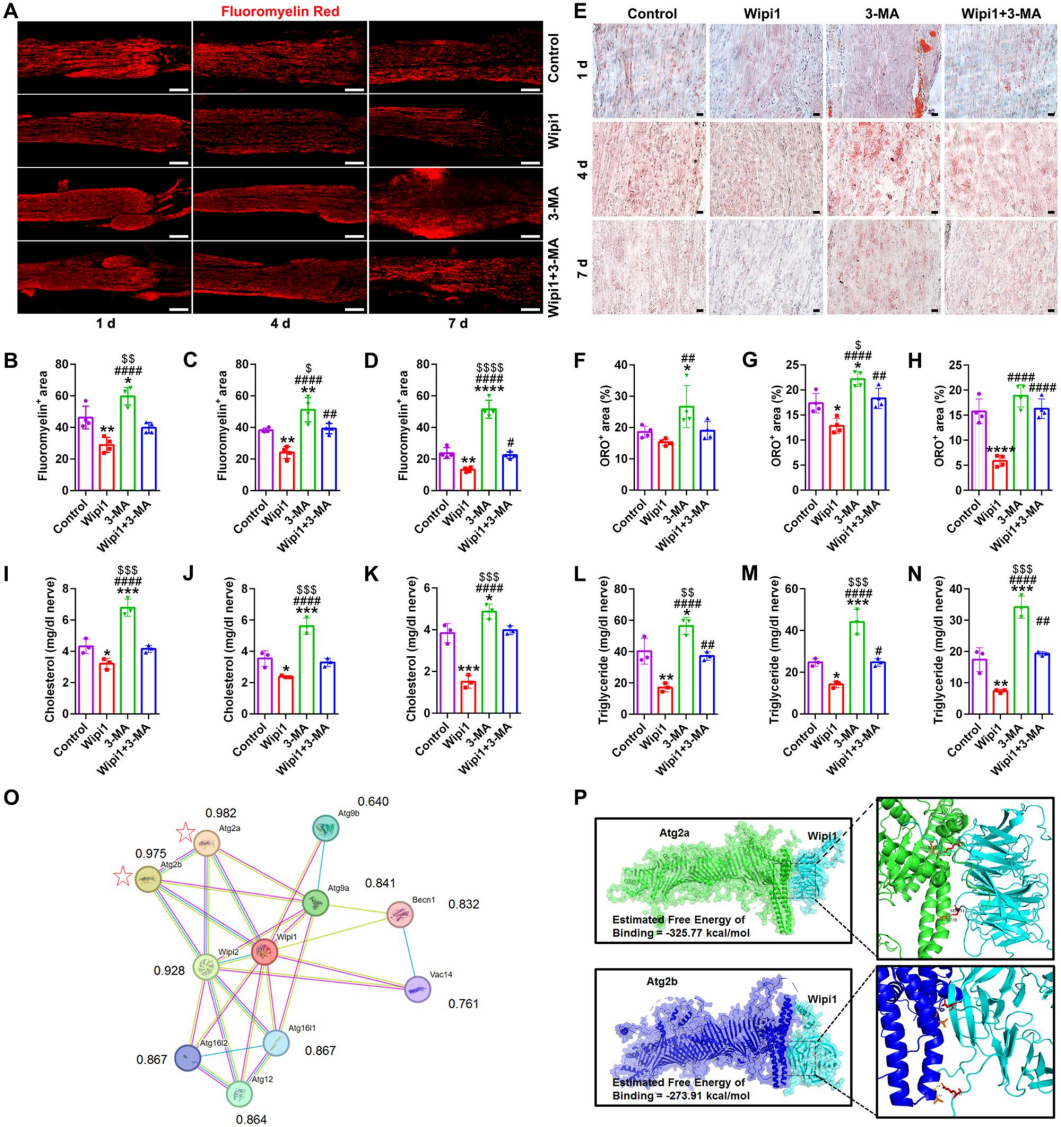
The regulatory effect of Wipi1 on myelin clearance and lipid metabolism. (**A**) Representative images of the fluoromyelin red staining at 1 d, 4 d and 7 d after injuries. Scale bar, 200 μm. (**B**–**D**) Histograms of the positive fluoromyelin area at 1 d, 4 d and 7 d after injuries, respectively, *n* ≥ 3. *, each group vs. control group. #, each group vs. Wipi1 group. $, each group vs. Wipi1 + 3-MA group. *, *P* < 0.05; **, *P* < 0.01; ****, *P* < 0.0001. #, *P* < 0.05; ##, *P* < 0.01; ####, *P* < 0.0001. $, *P* < 0.05; $$, *P* < 0.01; $$$$, *P* < 0.0001. (**E**) Representative images of the oil red O staining at 1 d, 4 d and 7 d after injuries. The lipid droplets appeared red. Scale bar, 20 μm. (**F**–**H**) Histograms of the percentage of positive oil red O area at 1 d, 4 d and 7 d after injuries, respectively, *n* = 3. *, *P* < 0.05; ****, *P* < 0.0001. ##, *P* < 0.01; ####, *P* < 0.0001. $, *P* < 0.05. (**I**–**K**) Histograms of the total cholesterol at 1 d, 4 d and 7 d after injuries, respectively, *n* = 3. *, *P* < 0.05; ***, *P* < 0.001. ####, *P* < 0.0001. $$$, *P* < 0.001. (**L**–**N**) Histograms of the TG at 1 d, 4 d and 7 d after injuries, respectively, *n* = 3. *, *P* < 0.05; **, *P* < 0.01; ***, *P* < 0.001. #, *P* < 0.05; ##, *P* < 0.01; ####, *P* < 0.0001. $$, *P* < 0.01; $$$, *P* < 0.001. (**O**) The protein interaction network of Wipi1. The *R* value indicated the degree of correlation. The pentagrams marked the molecules with the highest *R* values with Wipi1: Atg2a (*R* = 0.982) and Atg2b (*R* = 0.975). (**P**) Structure-based protein interaction interface of Wipi1 and Atg2. The binding free energy was estimated. The enlarged field on the right displaysed the amino acid binding site and the hydrogen bond interaction of Wipi1–Atg2 complex.

The direct effect of Wipi1 on myelin clearance including lipid metabolism was observed. The results showed that at all time points, myelin areas of the Wipi1 group were significantly reduced; and the inhibitor reversed the accelerated clearance of myelin debris (Figure 8A–H). To further study the role of lipid accumulation, the contents of THO and TG were detected. It was displayed that the THO and TG in the injured tissues were significantly decreased at different time points after Wipi1 treatment. The THO and TG in the other groups were significantly higher than those of the Wipi1 group, indicating that the Wipi1 treatment activated lipid catabolism in the injured tissues (Figure 8I–N). Possible interacting partners of Wipi1 were examined. The interaction network of 10 proteins was depicted (Figure 8O). Wipi1 protein was positively linked with the Atg2a (*R* = 0.982) and Atg2b (*R* = 0.975). Therefore, Wipi1 may play the regulatory role in lipid metabolism by interacting with Atg2. Next, the interaction between Wipi1 and Atg2a, Atg2b was analyzed by molecular docking (Figure 8P). It was revealed that Wipi1 probably bound to Gln 1677 and Arg 1738 of Atg2a in the model structure complex of the Wipi1 domain and the Atg2a linker domain, with an estimated free energy binding of −325.77 kcal/mol. Then Wipi1 formed a protein interaction interface with Atg2b and the important amino acid residues at the active site were Thr 1772 and Val 1786 with an estimated free energy binding of −273.91 kcal/mol. So the hydrogen bond interaction of amino acids at different sites contributed to the formation of Wipi1-Atg2 complex, thereby maintaining the stability of the dimer. These data clearly revealed that Wipi1 had a positive impact on peripheral myelin lipid catabolism with which Atg2 was a key molecule.

## Discussion

The chitosan artificial nerve graft with PLGA guiding fibers is an independently developed product in our laboratory, which is combined with other biomaterials or supporting cells to further construct tissue engineered nerves [42, 43]. A series of animal experiments, preclinical experiments and clinical trials have painted that the chitosan artificial nerves were able to effectively repair long-distance peripheral nerve defects [44–46]. Chitosan, as a natural biomaterial, not only possess good biocompatibility, but also excellent controllable degradation performance after implantation *in vivo*. How chitosan and its degradation products affect the local nerve regenerative microenvironment and even the overall environment of the body has always been one of the exploration directions. This is very important for guiding us to design and construct better artificial nerves and tissue engineered nerves [5]. COS, the main intermediate degradation products of chitosan, continuously communicate with local cells and tissues during nerve regeneration, directly affecting the regeneration microenvironment. Our previous studies illustrated that COS had the activity of regulating and improving the microenvironment, mainly in the regeneration stage. However, the clearance of myelin debris during the preparation stage is equally crucial for nerve regeneration [47–49]. COS have been revealed to regulate the macrophages and SCs. So does COS also affect Wallerian degeneration by regulating the two main cells involved in myelin clearance? Previous team studies did not focus on the impact of biomaterials in this stage of nerve regeneration, and there have been few other related studies. There is little research on biomaterial roles in Wallerian degeneration.

The sciatic nerve crush injury model, due to its faster and better self-regeneration process compared to the defect model, was selected to observe the promoting effect of COS, especially on the myelin clearing. Through the observation and evaluation of motor and sensory functions, regenerated nerve electrical signal transmission and tissue structure, COS significantly promoted peripheral nerve regeneration, which was consistent with our other studies [35, 38, 50]. However, the intervention with COS did not show the improvement in mechanical pain, which may be due to the relatively delayed recovery of nerve reinnervation to stress perception. Subsequently, the results indicated that COS played a role in accelerating the myelin clearance, and correspondingly, the myelin proteins also displayed the same trend of changes in a later period of time.

It is found that after peripheral nerve injury, SCs initially degrade the myelin sheath, and then, SCs recruit macrophages to the degenerated nerves by releasing various chemokines and cytokines to assist in clearing the degraded myelin [51]. In addition, endothelial cells also participate in myelin removal as an amateur role [52, 53]. The degradation of artificial nerve grafts after implantation requires a large amount of macrophages [54, 55]. Is the accelerating effect of COS on myelin clearance achieved through macrophages or SCs? Regardless of the ways of myelin breakdown, it ultimately relies on the action of hydrolytic enzymes in lysosomes. The expression of lysosome marker LAMP1 is induced after nerve injury and is associated with degraded myelin sheath fragments [56]. The role of SC autophagy in myelin clearance has become a focus of attention. After peripheral nerve injury, SCs themselves, or SCs cooperate with macrophages, or macrophages affect the autophagy and phagocytosis of SCs to clear myelin debris, thereby removing obstacles for nerve regeneration [24, 57, 58]. LC3B is a widely used autophagy marker that is localized on the autophagosome membrane. The experimental results indicated that as Wallerian degeneration progressed, COS significantly increased the number of lysosomes and autophagosomes in SCs, directly regulating the autophagy function and myelin clearance of SCs, which emerged early and continued after nerve injury. The TEM observation, the gold standard for autophagy detection [59], further clarified that COS increased the number of autophagosomes in SCs after nerve injury. Meanwhile, the intervention of COS had a relatively limited and delayed effect on the autophagy function of macrophages, only reflected in a limited time period of 1 d and 4 d after surgery. So SCs played a more important role in the autophagy, and COS had a regulatory effect on the occurrence of autophagy in myelin clearance, mainly by affecting SC autophagy.

The quantitative proteomics was used to further explore the mechanism of COS promoting myelin clearance. Annotation analysis demonstrated that a large number of differential proteins were related to lysosome and autophagy regulations. These differentially expressed proteins were enriched not only in stress responses, energy metabolism, neurodevelopment, angiogenesis and immune responses but also in biological processes and pathways such as lysosome, autophagy, Golgi apparatus, lipid metabolism and cytoskeleton. The results revealed the regulatory role and mechanism of COS on the process of myelin clearance from a molecular regulatory perspective, in which autophagy was an important aspect. Through the screening and validation of differential proteins, the expression of Wipi1 protein was significantly upregulated, indicating its key role in COS regulation of myelin clearance. In the top significantly upregulated proteins, the main regulatory role of Wipi1 directly point to autophagy compared to others. The Wipi1 protein family with conserved sequences was found to play an important role in regulating the autophagosome formation, and is an effector molecule of the autophagy initiation signal phosphoinositol [60, 61]. Moreover, the immunohistochemistry showed that COS upregulated expression of Wipi1 protein in SCs throughout the Wallerian degeneration mediating the autophagy of SCs and myelin clearance.

**Figure 9.**
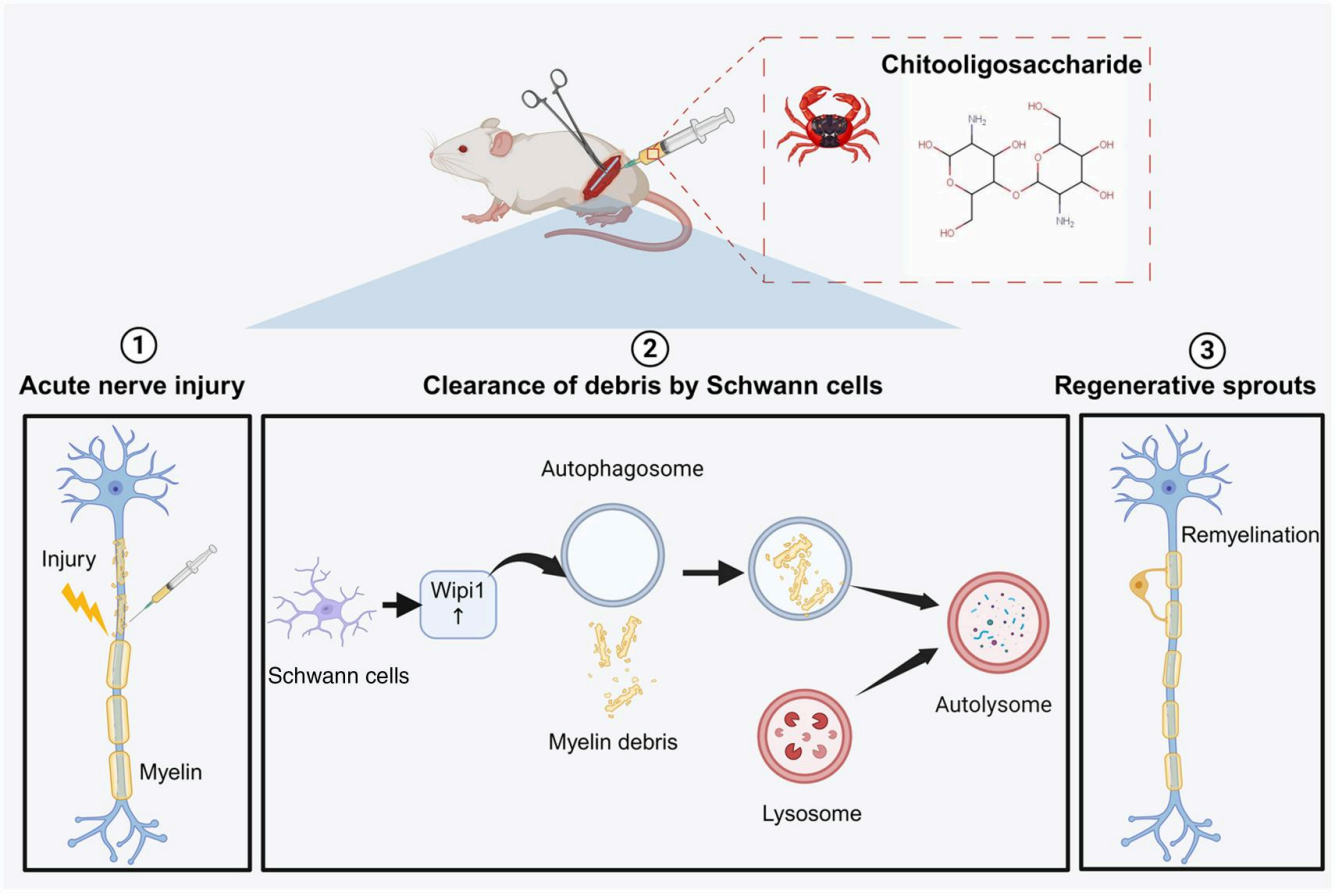
Chitooligosaccharides up-regulated the expression of Wipi1 protein in Schwann cells, mediated autophagy to remove myelin and promote nerve regeneration. With the occurrence of Wallerian degeneration, COS increased the number of lysosomes and autophagosomes in Schwann cells, promoting myelin clearance and nerve repair. Importantly, by upregulating the expression of Wipi1 in Schwann cells, this regulatory process effectively promotes the phagocytosis and degradation of myelin fragments, thus, shortening the myelin remodeling and axon growth, eventually contribute to the recovery process of injured peripheral nerve.

Finally, the experiment evaluated the effect of Wipi1 protein on promoting peripheral nerve regeneration. The Wipi1 protein comprehensively enhanced peripheral nerve regeneration including the sensory and motor function, electrophysiology and tissue structure. More importantly, the promoting effect of Wipi1 protein on nerve regeneration was directly related to its acceleration of myelin clearance involving lipid metabolism indicating its potential as a clinical repair target. Then, except for SFI, all other indicators displayed that the inhibitor of Wipi1 almost completely reversed the promoting effect of Wipi1 protein. The possible reasons for the difference in SFI were twofold: first, the impact of inhibitor injection itself; secondly, the regulatory role of other molecules in the functional recovery. In view of the previously reported role of Wipi1-Atg2 in autophagy and also the effect of interaction with Atg2 on lipid metabolism, we studied whether Wipi1 regulates lipid metabolism during myelin debris clearance under physiological conditions after peripheral nerve injury. Wipi1 promoted the metabolism of THO and TG by regulating ATG2, so that the lipid droplets of disintegrated myelin sheath could be quickly and effectively removed to clear the road for nerve regeneration. In sum, the Wipi1 protein played an important regulatory role in nerve regeneration. Our experiment depicted that chitosan and its degradation derivative COS not only have a positive neural regulatory effect during peripheral nerve regeneration, but also promote the clearance of myelin debris through Wipi1 mediated SC autophagy (Figure 9). Our research enriches the cognition of underlying logic behind the chitosan artificial nerve repairing nerve defects and deepens the understanding of communications between biomaterials and organisms. Our figures provide the new experimental evidence and perspectives for the targeted discovery and resolution of existing shortcomings of artificial nerves and tissue engineered nerves, as well as the application of biomaterials in design and construction of artificial tissues and organs in a beneficial and harm avoidance manner.

## Conclusion

Chitooligosaccharides accelerate myelin clearance by Wipi1 mediated Schwann cell autophagy promoting peripheral nerve regeneration. The research deepens the comprehensive understanding of the positive regulatory role of chitosan and chitooligosaccharides; and expands new contents and ideas for designing and constructing better tissue engineered nerves from the perspective of mutual communication and response between biomaterials and tissues.

## Supplementary Material

rbaf044_Supplementary_Data

## Data Availability

All data generated or analysed during this study are included in this published article and its supplementary information files. *Conflicts of interest statement*. None declared.
