# Supplementary material for: Chitooligosaccharides accelarate myelin clearance by Wipi1 mediated Schwann cell autophagy promoting peripheral nerve regeneration
Source: Regen Biomater. 2025 May 19;12:rbaf044. doi: 10.1093/rb/rbaf044 (PMC12466441; doi:10.1093/rb/rbaf044)
Supplement: rbaf044_Supplementary_Data [file rbaf044_Supplementary_Data.docx]

Responses to Reviewers:

Reviewer: 1

Comments to the Author

no further comments

Thank you for the comments!

Reviewer: 2

Comments to the Author

Most of the questions have been answered in the revised manuscript. There is one more question that needs clarification:

The explanation for the experiment design is accepted, but the description, i.e. “The crush injury was performed 3 minutes after injection,” differs from the picture on Page 72, which shows that crush injury occurred before the injection.

Thanks a lot for the comment! We apologize for the inconsistency caused by the imprecise description of the experimental details. The actual experimental procedure was indeed to crush the nerve just 3 minutes after the COS injection, which was the specific operational design. In terms of the logical essence of the experimental design, it still belongs to post injury treatment and does not belong to pre-protection. Then the figure 9 was modified and replaced. We will improve the rigorousness of this issue in similar experimental designs in the future. I hope our explanation and modification could address your concerns.

Review Editor

Comments to the Author:

This manuscript is of initial interest to the Board but will require revisions before the potential publication. Please submit a revised version which will be reviewed again.

Thank you for the comments! The question of the reviewer was responsed. The figure 9 was modified and replaced.
